# Stratified Care in Cognitive Behavioural Therapy: A Comparative Evaluation of Predictive Modelling Approaches for Individualized Treatment: La stratification des soins pour l’orientation vers une thérapie cognitivo-comportementale: une évaluation comparative des approches de modélisation prédictive pour un traitement individualisé

**DOI:** 10.1177/07067437241295635

**Published:** 2024-11-10

**Authors:** Margaret Jamieson, Andrew Putman, Marsha Bryan, Bojay Hansen, Phillip E. Klassen, Nicholas Li, Bethany McQuaid, David Rudoler

**Affiliations:** 1 Institute of Health Policy, Management, and Evaluation, 7938 University of Toronto, Toronto, Ontario, Canada; 2 25487 Ontario Shores Centre for Mental Health Sciences, Whitby, Ontario, Canada; 3 Faculty of Health Sciences, Ontario Tech University, Oshawa, Ontario, Canada; 4 Department of Psychiatry, 7938 University of Toronto, Toronto, Ontario, Canada

**Keywords:** depressive disorder, cognitive behavioural therapy, community mental health services, precision medicine, supervized machine learning, predictive models, trouble dépressif, thérapie cognitivo-comportementale, services communautaires de santé mentale, médecine de précision, supervisé apprentissage automatique, modèles prédictifs

## Abstract

**Objective:**

In response to high demand and prolonged wait times for cognitive behavioural therapy (CBT) in Ontario, Canada, we developed predictive models to stratify patients into high- or low-intensity treatment, aiming to optimize limited healthcare resources.

**Method:**

Using client records (*n* = 953) from Ontario Shores Centre for Mental Health Sciences (January 2017–2021), we estimated four binary outcome models to assign patients into complex and standard cases based on the probability of reliable improvement in Patient Health Questionnaire-9 (PHQ-9) and Generalized Anxiety Disorder-7 (GAD-7) scores. We evaluated two choices of cut-offs for patient complexity assignment: models at an ROC (receiver operating characteristic)-derived cut-off and a 0.5 probability cut-off. Final model effectiveness was assessed by assigning treatment intensity (high-intensity or low-intensity CBT) based on the combined performance of both GAD-7 and PHQ-9 models. We compared the treatment assignment recommendations provided by the models to those assigned by clinical assessors.

**Results:**

The predictive models demonstrated higher accuracy in selecting treatment modality compared to provider-assigned treatment selection. The ROC cut-off achieved the highest prediction accuracy of case complexity (0.749). The predictive models exhibited large sensitivity and specificity trade-offs (which influence the rates of patient assignment to high-intensity CBT) despite having similar accuracy statistics (ROC cut-off = 0.749, 0.5 cut-off = 0.690), emphasizing the impact of cut-off choices when implementing predictive models.

**Conclusions:**

Overall, our findings suggest that the predictive model has the potential to improve the allocation of CBT services by shifting incoming clients with milder symptoms of depression to low-intensity CBT, with those at highest risk of not improving beginning in high-intensity CBT. We have demonstrated that models can have significant sensitivity and specificity trade-offs depending on the chosen acceptable threshold for the model to make positive predictions of case complexity. Further research could assess the use of the predictive model in real-world clinical settings.

**Plain Language Summary Title:**

Stratified Care in Cognitive Behavioural Therapy: A Comparative Evaluation of Predictive Modeling Approaches for Individualized Treatment

## Plain Language Summary

In response to high demand and prolonged wait times for cognitive behavioral therapy (CBT) in Ontario, Canada, we developed predictive models to stratify patients into high- or low-intensity treatment, aiming to optimize limited healthcare resources.

We used client records from Ontario Shores Centre for Mental Health Sciences (January 2017–2021). The final model's effectiveness was assessed by assigning treatment intensity (high-intensity or low-intensity CBT) based on treatment success. Treatment success was determined using validated symptom scales. We compared the treatment assignment recommendations provided by the predictive model to those assigned by clinical assessors. Overall, our findings suggest that the predictive model has the potential to improve the allocation of CBT services by shifting incoming clients with milder symptoms of depression to low-intensity CBT, with those at highest risk of not improving beginning in high-intensity CBT.

## Résumé

**Objectif:**

En réponse à la forte demande et aux temps d’attente prolongés pour recevoir une thérapie cognitivo-comportementale (TCC) en Ontario, au Canada, nous avons élaboré des modèles prédictifs pour stratifier les patients dans un traitement de haute ou de faible intensité, dans le but d’optimiser les ressources limitées en soins de santé.

**Méthode:**

En utilisant les dossiers des clients (*n* = 953) du Ontario Shores Centre for Mental Health Sciences (janvier 2017–2021), nous avons estimé quatre modèles de résultats binaires pour classer les patients en cas complexes et en cas standard sur la base de la probabilité d’une amélioration fiable des scores mesurés par le Patient Health Questionnaire-9 (PHQ-9) et l’échelle Generalized Anxiety Disorder-7 (GAD-7). Nous avons évalué deux choix de seuils pour l’attribution de la complexité du patient : des modèles à un seuil dérivé de la fonction d’efficacité du récepteur et un seuil de probabilité de 0,5. L’efficacité du modèle final a été évaluée en attribuant l’intensité du traitement (TCC de haute intensité ou de faible intensité) en fonction de la performance combinée des modèles GAD-7 et PHQ-9. Nous avons comparé les recommandations d’attribution de traitement fournies par les modèles à celles attribuées par les évaluateurs cliniques.

**Résultats:**

Les modèles prédictifs ont révélé une plus grande précision quant au choix de la modalité de traitement par rapport au choix du traitement attribué par le prestataire de soins. Le seuil dérivé de la fonction d’efficacité du récepteur a obtenu la plus haute précision de prédiction de la complexité des cas (0,749). Les modèles prédictifs présentaient des compromis importants entre sensibilité et spécificité (qui influencent les taux d’affectation des patients à une TCC de haute intensité) malgré des statistiques de précision similaires (seuil dérivé de la fonction d’efficacité du récepteur = 0,749, seuil de probabilité de 0,5 = 0,690), ce qui met en évidence les conséquences du choix des seuils lors de la mise en œuvre des modèles prédictifs.

**Conclusions:**

Dans l’ensemble, nos constatations laissent penser que le modèle prédictif peut améliorer l’attribution des services de TCC en orientant les nouveaux clients présentant des symptômes de dépression plus légers vers une TCC de faible intensité, les clients les plus susceptibles de ne pas s’améliorer se voyant offrir dès le début une TCC de haute intensité. Nous avons montré que les modèles peuvent présenter des compromis importants entre sensibilité et spécificité en fonction du seuil acceptable choisi pour que le modèle fasse des prédictions positives sur la complexité du cas. D’autres recherches pourraient évaluer l’utilisation du modèle prédictif dans des contextes cliniques réels.

## Introduction

High demand and long wait times for community mental health treatment in Ontario have made it necessary to improve access to timely and therapeutically appropriate care for individuals experiencing anxiety and mood disorders.^1,2^ Publicly funded treatments and support networks for these disorders have historically been difficult to access. Despite expansions to psychotherapy and cognitive behavioural therapy (CBT) programs in the province, there are still issues meeting demand.^3,4^

Programs such as the Ontario Structured Psychotherapy (OSP) program and other outpatient treatment options have worked to address the shortage of adequate psychotherapy services in Ontario. At Ontario Shores Centre for Mental Health Sciences,^5^—a mental health and addictions facility located in Whitby, Ontario—patients can receive outpatient treatment at an intensity best suited to their clinical needs. One way to assign treatment intensity is through a process known as stepped care, which involves starting patients at low-intensity CBT (LICBT), such as self-guided formats, and moving up to higher-intensity treatment (HICBT) if symptoms do not improve. A key component of optimizing care delivery in both LICBT and HICBT is ensuring that patients are provided with sufficient and appropriate treatment to improve their symptoms, while minimizing unnecessary treatment that might deny other patients the opportunity to receive therapy.

An alternative to stepped care is the use of predictive modelling techniques and precision medicine to triage patients into LICBT and HICBT based on their likelihood of experiencing clinical improvement—a method called stratified care. Stratified care involves grouping patients according to baseline clinical and sociodemographic characteristics to identify those that will respond to specific types of treatment.^6–8^ A recent meta-analysis of papers (*n* = 24) predicting CBT response found that classifying methods were able to distinguish responders from non-responders based on pre-treatment characteristics with a pooled positive predictive accuracy of 74.0%.^9^ Another systematic review (*n* = 89) compared and validated available research on the use of predictive models to support diagnostic and treatment decisions, finding several data-driven predictive tools that could be used to support psychiatric treatment decisions.^10^ Research into the Improving Access to Psychological Therapies (IAPT) program in the UK demonstrated that using a predictive model to classify patients at intake into LICBT and HICBT treatment is cost-effective relative to stepped care and improves overall patient outcomes.^11,12^ Given the demonstrated improvements in the efficiency of care delivery, this approach could benefit other regions facing high demand and long wait times for outpatient psychotherapies.

In this study, we aim to replicate, where possible, this approach in a stepped-care outpatient psychotherapy program in Ontario, Canada. However, due to differences in the local context and data collection, we could not apply existing predictive models. Instead, we retrained predictive models using local hospital-level data to enhance client intake for outpatient psychotherapy services in Ontario. We also add to the literature by investigating how model sensitivity and specificity changes with the empirical cut-offs used to assign patient complexity. To the best of our knowledge, this represents the first attempt to compare and validate different cut-offs using empirical data in this context. The outcomes of this investigation will not only guide the process of client intake and triage at our study hospital but may also serve as a valuable reference for similar clinical contexts employing comparable datasets.

## Method

### Setting and Intervention

Our study is set at Ontario Shores Centre for Mental Health Sciences. Outpatient psychotherapy at Ontario Shores is provided to qualifying clients with symptoms of anxiety and mood disorders via three different modalities according to their level of clinical need. Electronic CBT (eCBT) at Ontario Shores is delivered alongside a regulated clinical professional and involves online learning modules and virtual coaching. Alternatively, structured group therapy and one-on-one structured therapy were also available in addition to eCBT. We chose to include client data from the period leading up to the COVID-19 pandemic, and data collected during the pandemic itself to adequately account for any changes to demand mental health treatment. This study covers treatment received between 1 January 2017 and 31 December 2021. Ethics approval for this study was provided by Ontario Shores REB (file #: 22-025-D).

### Data Handling and Collection

We conducted a retrospective chart review based on data from Ontario Shores’ electronic medical record (EMR) data. Data records were initially collected by healthcare providers at the hospital, stored in the EMR, linked using unique identifiers, and anonymized prior to our analysis. Records included both clinical and diagnostic information for clients receiving treatment at the Anxiety and Mood Disorder Clinic at Ontario Shores, including assessments, consultations, and data collected at psychotherapy sessions.

### Participants

All clients with non-confidential EMRs completing CBT treatment at the Anxiety and Mood Disorder Clinic between 1 January 2017 and 31 December 2021 were included in this study, with the date of the first pre-session evaluation chosen as the index date for inclusion. We excluded clients who did not have recorded Patient Health Questionnaire-9 (PHQ-9) or Generalized Anxiety Disorder-7 (GAD-7) scores for at least two sessions, including their initial session, and those who did not complete treatment. For eCBT clients, treatment was considered complete at 98 days following the admission date, and for group and individual CBT clients, the treatment completion cut-offs were set at 147 days. These benchmarks were set by the Anxiety and Mood Disorder Clinic at Ontario Shores, which also classified treatment as being complete if a client showed reliable improvement before their treatment cut-off was reached. After reporting descriptive statistics, missing data from the sociodemographic variables for employment status (6%), living arrangement (3%), and the neighborhood-level descriptive variables (5%) were imputed. Following Delgadillo et al.,^12^ we used single imputation of each variable's mean probability score.

### Outcome Measures

#### Clinical Outcomes

Following Delgadillo et al.,^12^ our first outcomes of interest are the binary indicators of the client having experienced a reliable and clinically significant improvement (RCSI) in PHQ-9 or GAD-7 scores after having completed treatment. RCSI was determined separately for the PHQ-9 and GAD-7 scores for each client. A classification of having achieved RCSI for the PHQ-9 was determined by a client's initial PHQ-9 score being ≥10 (indicating clinical significance of depression symptoms) and having the difference between their initial and last recorded scores be ≥6 points. RCSI for the GAD-7 was determined by a client having an initial GAD-7 score of ≥8 (which indicates the likely presence of an anxiety disorder) and the difference between initial and last recorded GAD-7 scores ≥5.^13,14^

#### Actual Treatment Intensity

Individuals from this sample had been initially assigned into high or low CBT treatment intensity streams (HICBT or LICBT) in consultation with the clinician who was performing their intake. Using definitions provided by clinical teams at Ontario Shores, LICBT was defined as eCBT or group CBT, while HICBT involved one-on-one CBT sessions. If a client who was initially assigned to the LICBT stream was not responding well a clinician could step up that clients care by moving them into HICBT after the initial treatment intensity assignment (stepped care).

### Predictive Variables

In addition to initial PHQ-9 and GAD-7 scores, EMR data provided us with clinical and sociodemographic characteristics for the sample including client age, biological sex, living arrangement, and employment status. Using postal code and the Ontario Marginalization Index we obtained neighbourhood ethnic concentration, residential instability, material deprivation, and dependency.^15^

### Analytic Approach

In keeping with the approach used by Delgadillo et al.,^12^ we generated and validated a predictive model of the stratified care pathway based on our available data. Our analysis was carried out in the following stages, replicating stages 2–4 for PHQ-9 and GAD-7 scores.

#### Data Cleaning and Preparation

Following the exclusion of clients who did not meet the inclusion criteria, data were structured so that each observation was a single treatment episode for a single client. Then we split the dataset 50–50 into training and test samples to train our model, and then validate with the remaining test observations.

#### Identification of RCSI Likelihoods and Classification Method to Identify “Complex” Cases

Two linear regression models (one predicting RCSI of the PHQ-9 scores and the other predicting RCSI of the GAD-7 scores) were created from the training dataset using Least Absolute Shrinkage and Selection Operator (LASSO) penalization for variable selection as well as reducing the degree of model overfitting of the training data. The LASSO penalization process introduces a λ term that favours coefficients approaching zero, mitigating overfit and enhancing the generalizability of prediction equations compared to regression models using the standard least squares penalization. We used the .632 bootstrap resampling method to create 1,000 iterations of each model using λ values between 10^−4^ and 10^−1^, from which the optimal λ value for each model was selected.^16^ We used the model estimates to generate PHQ-9 and GAD-7 RCSI probability scores for each patient, which could then be incorporated into a predictive measure of their case complexity. Finally, we generated receiver operating characteristic (ROC) curves for the PHQ-9 and GAD-7 probability scores separately.

#### Determine Empirical Cut-Offs to Optimize Sensitivity and Specificity for Each Predictive Model

The ROC curves were used to select two cut-off points for each model, a cut-off point that maximized combined specificity and sensitivity, and a standard 0.5 cut-off (50% likelihood of predicted RCSI) that is typical of logistic regression for comparison.

#### Model Predicting RCSI

Next, we utilized the LASSO predictive models with the two selected cut-off thresholds on the test dataset to predict whether clients would achieve RCSI in their PHQ-9 or GAD-7 scores separately and then generated confusion matrices and related statistics comparing the predicted RCSI in PHQ-9 or GAD-7 at each threshold with the actual RCSI in PHQ-9 or GAD-7 seen in the sample.

#### Comparison of Model with Clinician Assignment of Treatment Intensity

Finally, we used the models to generate predictions of RCSI PHQ-9 or GAD-7 classifications on the entire dataset (*n* = 953). Then those predictions were used to further predict client case complexity. Using Delgadillo et al's definition of complex cases (C*x*), clients were classified as having been predicted to be complex if their initial score met the thresholds of clinical significance for the PHQ-9 and GAD-7 measures, and both the PHQ-9 model and the GAD-7 model predicted that the client symptoms would not meet the threshold for RCSI. Thus, a client who is predicted to achieve RCSI in either or both predictive models would be deemed a standard case (St). Following Delgadillo's definition of case complexity, clients who were predicted to be C*x* were assigned to HICBT, and clients predicted to be St were assigned to LICBT. This allowed us to use confusion matrices to directly compare the model's predictions with the CBT treatment intensity that each client was assigned by the admitting clinician.

## Results

### Client Population

After data aggregation and cleaning, there were 1,718 patients with valid records. After excluding clients with less than two recorded scores, we had 953 clients who met the initial inclusion and exclusion criteria. Summary details comparing the sample and those who did not meet the initial exclusion criteria are in Table A1 in the supplementary materials. Living arrangement was the only baseline variable used in the predictive model that had a standardized mean difference greater than 0.2. Full summary details for the sample are described in Table 1.

**Table 1.** Summary of Included Sample.

	Total sample (*n* = 953)
Sociodemographic characteristics	
Age at admission (mean, SD)	34.6 (13.11)
Sex (%)	
Male	295 (31%)
Female	658 (69%)
Employment group (%)	
Employed	418 (44%)
Not employed	475 (50%)
Missing	60 (6%)
Living arrangement (%)	
By self	115 (12%)
With children	60 (6%)
With non-family	32 (3%)
With other relatives	77 (8%)
With parents	287 (30%)
With spouse/partner	171 (18%)
With spouse/partner and roommate	181 (19%)
Prefer not to answer/don’t know/missing	30 (3%)
Instability quantile (%)	
1st	233 (24%)
2nd	186 (20%)
3rd	198 (21%)
4th	152 (16%)
5th	133 (14%)
Missing	51 (5%)
Deprivation quantile (%)	
1st	149 (16%)
2nd	253 (26%)
3rd	197 (21%)
4th	146 (15%)
5th	157 (16%)
Missing	51 (5%)
Dependency quantile (%)	
1st	300 (31%)
2nd	187 (20%)
3rd	168 (18%)
4th	138 (14%)
5th	109 (11%)
Missing	51 (5%)
Ethnic concentration quantile (%)	
1st	85 (9%)
2nd	168 (18%)
3rd	248 (26%)
4th	248 (26%)
5th	153 (16%)
Missing	51 (5%)
Clinical characteristics	
Actual treatment intensity type (%)	
High-intensity CBT (one-on-one)	347 (36%)
Low-intensity and high-intensity CBT (stepped care)	62 (7%)
Low-intensity CBT (eCBT or group)	544 (57%)
First GAD-7 score (mean, SD)	13.1 (5.06)
Last GAD-7 score (mean, SD)	8.8 (5.38)
First PHQ-9 score (mean, SD)	15.0 (5.96)
Last PHQ-9 score (mean, SD)	10.1 (6.07)
GAD-7 RCSI (%)	
Below clinical threshold (initial score < 8)	161 (17%)
No RCSI (initial score ≥ 8 and initial – last score difference < 5)	362 (38%)
RCSI (initial score ≥ 8 and initial – last score difference ≥ 5)	430 (45%)
PHQ-9 RCSI (%)	
Below clinical threshold (initial score < 10)	186 (19%)
No RCSI (initial score ≥ 10 and initial – last score difference < 6)	390 (41%)
RCSI (initial score ≥ 10 and initial – last score difference ≥ 6)	377 (40%)
Case complexity (%)	
Complex (no RCSI for PHQ-9 and GAD-7)	238 (25%)
Standard (all else)	715 (75%)

*Note.* CBT = cognitive behavioural therapy; RCSI = reliable and clinically significant improvement.

Clients tended to be female (69%) and 35 years (SD = 13.11) old on average. Fifty-seven percent received LICBT, 7% received stepped care, and 36% received HICBT. Nearly half of the clients in our sample experienced RCSI in their GAD-7 (45%) and PHQ-9 scores (40%) at the completion of their treatment. For the remaining analysis, we randomly divided this sample into training (*n* = 477) and test (*n* = 476) samples for initial model creation.

### Predicting Case Complexity

#### Predicting RCSI for PHQ-9 and GAD-7

The LASSO model predicting RCSI in PHQ-9 scores retained four non-zero predictive variables after λ penalization (initial PHQ-9 score, employment status, instability, and living with their spouse and roommate), and the LASSO model predicting RCSI in GAD-7 scores retained two predictive variables (initial GAD-7 score and living with non-family members). The model coefficients for multivariate LASSO linear regression models with optimal λ values using training dataset are presented in Table A2 in the supplementary materials.

Next, we generated ROC curves for analysis based on the LASSO models. We conducted ROC analysis to determine the optimal cut-offs for balancing sensitivity and specificity for each model in both the training and test datasets. The graphical results from this process are shown in Figures A1–A4 in the supplementary materials. We found that our LASSO models retained their predictive accuracy when applied to our test sample, with area under the curve (AUC) measures of 0.746 (95% CI 0.703–0.789) for the PHQ-9 model, and 0.747 (95% CI 0.704–0.791) for the GAD-7 model. Given the specificity–sensitivity imbalance shown in both model's ROC analyses, further testing of each model has been conducted with two cut-offs: a ROC-generated cut-off which maximizes the combined sensitivity and specificity, and a threshold of 0.50 cut-off representative of typical logistic regression methodology. Additional confusion matrix metrics comparing the 0.5 and ROC cut-offs for each intermediate model are reported in Table A3 in the supplementary materials. The intermediate predictive models combining the LASSO optimizations and these cut-offs will be referred to as the PHQ0.5, PHQ-ROC, GAD-0.5, and GAD-ROC, respectively.

To further assess internal validity of the LASSO models, we used the models (which were generated using the training dataset) to predict RCSI PHQ-9 and RCSI GAD-7 with the test sample (Table A4 in the supplementary materials). Odds ratios (OR) for predicting RCSI PHQ-9 or GAD-7 in the test dataset were calculated for both models, with all models showing significant predictive validity. The PHQ-0.5 predictive model had an OR of 3.15 (95% CI 2.14–4.69) on the test dataset, the PHQ-ROC an OR of 6.06 (95% CI 3.99–9.38), the GAD-0.5 an OR of 3.68 (95% CI 2.49–5.49), and the GAD-ROC an OR of 7.68 (95% CI 4.93–12.30). Our intermediate prediction models of whether a client achieved RCSI were able to identify clients with worse prognoses in the test sample.

#### Assigning Predicted Case Complexity

The four intermediate models’ RCSI predictions were used to classify clients in the entire dataset (*n* = 953) into C*x* and standard cases (St). Using Delgadillo et al.'s definition of C*x*, clients were assigned to C*x* if they were not predicted to achieve RCSI for either PHQ-9 or GAD-7, with all other scenarios being classified as St. The naming convention of the two cut-off options from the intermediate models (ROC and 0.5) are referred to as C*x*-ROC and C*x*-0.5, respectively.

### Comparison of Model with Clinician-Assigned Treatment

To allow for easier comparison between predicted and actual CBT treatment intensity, cases that were classified as C*x* by the predictive models were assigned a predicted appropriate treatment intensity of HICBT and St cases were assigned to LICBT using the entire dataset (*n* = 953). Table 2 is a frequency table representing the predicted and actual complexity assignments for the full dataset based on the actual treatment intensity assigned, the 0.5 cut-off, and the ROC cut-off predictive models.

**Table 2.** Frequency of Case Complexity Predicted by the C*x* Model and Actual CBT Intensity Received Using 0.5 and ROC Cut-Offs.

		Actual CBT intensity		
		C*x*	St	Total
Actual treatment	Pred. C*x* (HICBT)	97	250	347
	Pred. St (LICBT)	141	465	606
	Total	238	715	
C*x* model: 0.5 cut-off	Pred. Cx	100	157	257
	Pred. St	138	558	696
	Total	238	715	
C*x* model: ROC cut-off	Pred. C*x*	25	26	51
	Pred. St	213	689	902
	Total	238)	715)	

*Note.* CBT = cognitive behavioural therapy; C*x* = complex case; HICBT = high-intensity cognitive behavioural therapy; LICBT = low-intensity cognitive behavioural therapy; St = standard.

Confusion matrices comparing the predicted and actual CBT treatment intensity assigned with having not achieved RCSI for neither PHQ-9 nor GAD-7 (complex case definition) are presented in Table 3. Both predictive model cut-offs demonstrated higher accuracy than the clinician-assigned treatment intensities, achieving the highest accuracy (.749) using the ROC cut-off point. The clinician-assigned treatment had higher sensitivity (.408) than the ROC cut-off point, but lower sensitivity than the 0.5 cut-off (.420). The clinician-assigned treatment had the lowest specificity (.650), whereas the ROC-based cut-off model had the highest specificity (.964).

**Table 3.** Comparison of the Actual Treatment Assignment and Model-Derived Predictions with Actual Case Complexity.

Statistic	Actual intensity	C*x* model: 0.5 cut-off	C*x* model: ROC cut-off
Accuracy	.590	.690	.749
Sensitivity (true positive rate)	.408	.420	.105
Specificity (true negative rate)	.650	.780	.964

*Note.* C*x* = complexity.

## Discussion

The results of our study highlight the effectiveness of predictive modelling in optimizing the allocation of CBT treatment intensities. Both cut-off points for the predictive models exhibited notably higher accuracy compared to clinician-assigned treatment intensity, indicating the model's capacity to predict the patients whose symptoms were most likely to not achieve RCSI in either the PHQ-9 or GAD-7. Notably, the ROC-based and 0.5 cut-offs for the model demonstrated large trade-offs between sensitivity and specificity despite having similar accuracy statistics. This means that the complexity model's cut-off threshold could be fine-tuned to meet a wide range of desired sensitivity/specificity trade-offs while maintaining similar overall accuracy. This ability to adjust thresholds is of particular importance since the potential impacts of a higher sensitivity (more truly C*x* clients correctly identified as C*x*) are not necessarily equivalent to the impacts of a lower specificity (fewer truly St cases correctly identified as St), which is an implicit assumption of the overall accuracy statistic.^17^ While judgements about the relative importance of sensitivity and specificity will be context-specific, reporting on them can provide valuable insights to decision-makers who will have to weigh the consequences of these trade-offs.

Nonetheless, these findings suggest the current allocation of psychotherapy resources at Ontario Shores may not be optimized for identification and prioritization of clients who are at highest risk of not achieving clinically significant improvements in their depression or anxiety symptoms. Baseline symptoms of depression and anxiety were found to be important predictors of treatment response. Similar findings have been found in other studies of predictive models for treatment response in depression.^12,18^ With an accuracy rate of 74.9% (ROC-based) and 69% (0.5-based), the accuracy of our methods is fairly comparable to a selection of 12 validated predictive psychiatric models (ranging from 66% to 92%).^10^ Our ROC-based accuracy rate is similar to the pooled positive predictive accuracy rate found in a recent meta-analysis (74.9% compared with 74.0%).^9^ When compared with Delgadillo et al., our accuracy rates are also similar. Delgadillo's ROC-based methods had accuracy rates (as measured by AUC statistics) of 67% and 74% for PHQ-9 and GAD-7 models, respectively.^12^ Our choice of included sociodemographic and clinical variables in our predictive model is also validated by the wider literature base. A systematic review of 89 models found that age, sex/gender, race/ethnicity, functional level, traumatic experiences, and family history of psychiatric treatment were important determinants in many of these causal analyses, as we also found in our treatment of these variables.^10^

Although our study does highlight the potential of using predictive modelling to allocate mental health treatment based on diagnostic factors, there are some important limitations. First, we had a limited sample of patients (*n* = 953), which is further subject to sampling bias due to the necessity of having two measurements for both the PHQ-9 and GAD-7 to assess RCSI. However, most observed baseline characteristics were balanced between the excluded and included samples (see Table A1 in the supplementary materials). In addition, we used single imputation to retain small proportions (≤6%) of missing data on predictor variables. We note that this could have led to an underestimate of the variance for these variables. Second, we lacked the broader range of predictors used in previous studies to arrive at their model of case complexity, such as the results of screening tests for personality disorders, and social adjustment scales. It is possible we are missing valuable data that could have improved the accuracy of our model predictions. Third, prediction models cannot account for all elements in a patient's journey. There are likely patients for whom stepped care will always be an optimal option if, for example, the severity of their symptoms is not completely captured by PHQ-9 scores. For these patients, there still needs to be a means for them to be stepped up to accessing high-intensity services even if they have initially been assigned low-intensity care. Additionally, predictive models do not have the clinical experience or instincts of front-line clinicians. Predictive models are helpful tools, but they cannot replace clinicians in their ability to allocate treatment. Fourth, our chosen method of patient classification is also one of several options, with other studies classifying patients into subgroups using centroid-based, k-means, and density-based cluster methods.^19^ Further research is needed to assess the trade-offs of using different modelling and estimation approaches. Fifth, the implementation of predictive models such as the one described in this paper is likely context-specific. Thus, it is possible that our results will not be transferable to other outpatient settings where contextual factors like care protocols (e.g., dosage) and patient populations vary. Further research could externally validate these models in similar clinical settings.

Despite these limitations, our predictive models suggest that there is promise in using routinely collected data to generate more optimal allocation of scarce mental health services. This study also highlights the trade-offs that can result from using different cut-off points for patient complexity in CBT. Our results suggest that purely aiming to optimize accuracy is not the best approach for all scenarios. Therefore, we suggest that decision-makers involved in the administration of predictive models and stakeholders impacted by these models (e.g., clinicians, patients, and family members) be involved in the evaluation of these potential trade-offs and in the selection of cut-offs that best match care priorities and resource availability. To advance the use of predictive models in clinical settings, we recommend that they be continually updated with new data and reevaluated. They should also be tested in other clinical settings to assess their external validity. The feasibility of translating models from one setting to another will depend on the availability of comparable clinical data. In addition, we recommend that predictive models be thoroughly evaluated prior to full implementation to assess their intended and unintended impacts on patient health outcomes. Economic evaluations should also be completed to assess enhancements in care efficiency.

## Supplemental Material

sj-docx-1-cpa-10.1177_07067437241295635 - Supplemental material for Stratified Care in Cognitive Behavioural Therapy: A Comparative Evaluation of Predictive Modelling Approaches for Individualized Treatment: La stratification des soins pour l’orientation vers une thérapie cognitivo-comportementale: une évaluation comparative des approches de modélisation prédictive pour un traitement individualisé Supplemental material, sj-docx-1-cpa-10.1177_07067437241295635 for Stratified Care in Cognitive Behavioural Therapy: A Comparative Evaluation of Predictive Modelling Approaches for Individualized Treatment: La stratification des soins pour l’orientation vers une thérapie cognitivo-comportementale: une évaluation comparative des approches de modélisation prédictive pour un traitement individualisé by Margaret Jamieson, Andrew Putman, Marsha Bryan, Bojay Hansen, Phillip E. Klassen, Nicholas Li, Bethany McQuaid and David Rudoler in The Canadian Journal of Psychiatry
