# Supplementary material for: Stratified Care in Cognitive Behavioural Therapy: A Comparative Evaluation of Predictive Modelling Approaches for Individualized Treatment: La stratification des soins pour l’orientation vers une thérapie cognitivo-comportementale: une évaluation comparative des approches de modélisation prédictive pour un traitement individualisé
Source: Can J Psychiatry. 2024 Nov 10;69(12):880–7. doi: 10.1177/07067437241295635 (PMC11562943; doi:10.1177/07067437241295635)
Supplement: sj-docx-1-cpa-10.1177_07067437241295635 - Supplemental material for Stratified Care in Cognitive Behavioural Therapy: A Comparative Evaluation of Predictive Modelling Approaches for Individualized Treatment: La stratification des soins pour l’orientation vers une thérapie cognitivo-comportementale:  [file sj-docx-1-cpa-10.1177_07067437241295635.docx]

# Supplementary Materials

**Table A1:** Demographic characteristics comparison of study sample and those who did not meet the inclusion criteria.

|  | < 2 Measures [Excluded] | >= 2 Measures [Sample] | SMD |
| --- | --- | --- | --- |
| n | 765 | 953 |  |
| Sex (%) |  |  | 0.068 |
| Female | 504 (65.9) | 658 (69.0) |  |
| Male | 261 (34.1) | 295 (31.0) |  |
| Age at Admission (mean (SD)) | 33.58 (13.09) | 34.65 (13.11) | 0.082 |
| Employment Group (%) |  |  | 0.123 |
| Employed | 295 (38.6) | 418 (43.9) |  |
| Not Employed | 428 (55.9) | 475 (49.8) |  |
| Unknown/Missing | 42 (5.5) | 60 (6.3) |  |
| Living Arrangement (%) |  |  | 0.222 |
| By Self | 88 (11.5) | 115 (12.1) |  |
| With Child[ren] | 52 (6.8) | 60 (6.3) |  |
| With Non-Family | 22 (2.9) | 32 (3.4) |  |
| With Other Relatives | 58 (7.6) | 77 (8.1) |  |
| With Parent[s] | 230 (30.1) | 287 (30.1) |  |
| With Spouse/Partner | 109 (14.2) | 171 (17.9) |  |
| With Spouse/Partner & Roommate | 147 (19.2) | 181 (19.0) |  |
| Prefer Not to Answer/Don't Know/Missing | 59 (7.7) | 30 (3.1) |  |
| Instability Quantile (%) |  |  | 0.081 |
| 1st | 183 (23.9) | 233 (24.4) |  |
| 2nd | 150 (19.6) | 186 (19.5) |  |
| 3rd | 142 (18.6) | 198 (20.8) |  |
| 4th | 142 (18.6) | 152 (15.9) |  |
| 5th | 106 (13.9) | 133 (14.0) |  |
| Unknown/Missing | 42 (5.5) | 51 (5.4) |  |
| Deprivation Quantile (%) |  |  | 0.059 |
| 1st | 122 (15.9) | 149 (15.6) |  |
| 2nd | 184 (24.1) | 253 (26.5) |  |
| 3rd | 161 (21.0) | 197 (20.7) |  |
| 4th | 122 (15.9) | 146 (15.3) |  |
| 5th | 134 (17.5) | 157 (16.5) |  |
| Unknown/Missing | 42 (5.5) | 51 (5.4) |  |
| Dependency Quantile (%) |  |  | 0.074 |
| 1st | 216 (28.2) | 300 (31.5) |  |
| 2nd | 162 (21.2) | 187 (19.6) |  |
| 3rd | 143 (18.7) | 168 (17.6) |  |
| 4th | 114 (14.9) | 138 (14.5) |  |
| 5th | 88 (11.5) | 109 (11.4) |  |
| Unknown/Missing | 42 (5.5) | 51 (5.4) |  |
| Ethnic Concentration Quantile (%) |  |  | 0.078 |
| 1st | 77 (10.1) | 85 (8.9) |  |
| 2nd | 145 (19.0) | 168 (17.6) |  |
| 3rd | 207 (27.1) | 248 (26.0) |  |
| 4th | 182 (23.8) | 248 (26.0) |  |
| 5th | 112 (14.6) | 153 (16.1) |  |
| Unknown/Missing | 42 (5.5) | 51 (5.4) |  |
| Initial Treatment Intensity (%) |  |  | 0.329 |
| High Intensity CBT | 402 (52.5) | 347 (36.4) |  |
| Low Intensity CBT | 363 (47.5) | 606 (63.6) |  |
| Has First GAD-7 Score (%) |  |  | 0.859 |
| Yes | 559 (73.1) | 953 (100.0) |  |
| No | 206 (26.9) | 0 (0.0) |  |
| First GAD-7 Score (mean (SD)) | 12.45 (5.63) | 13.05 (5.06) | 0.113 |
| Has First PHQ-9 Score = No (%) |  |  | 0.601 |
| Yes | 648 (84.7) | 953 (100.0) |  |
| No | 117 (15.3) | 0 (0.0) |  |
| First PHQ-9 Score (mean (SD)) | 15.45 (6.32) | 14.99 (5.96) | 0.075 |

**Table A2:** Regression coefficients for optimal λ value LASSO linear regressions predicting RCSI in PHQ-9 & GAD-7.

| ***Outcome Variable*** | RCSI PHQ-9 | RCSI GAD-7 |
| --- | --- | --- |
| *Optimal λ value* | 0.009 | 0.1 |
| **Predictive Variable** |  |  |
| Initial PHQ-9 Score | 0.199 | - |
| Initial GAD-7 Score | -0.031 | .097 |
| Age | 0.003 | - |
| Sex | - | - |
| Employment Status | 0.352 | - |
| Instability Index | -0.148 | - |
| Deprivation Index | 0.054 | - |
| Dependency Index | 0.002 | - |
| Neighbourhood Ethnic Concentration | -0.030 | - |
| Living Arrangement: |  |  |
| By Self | 0.028 | - |
| With Children | 0.345 | - |
| With Non-Family | -0.202 | - |
| With Other Relatives | 0.422 | - |
| With Parent[s] | - | - |
| With Spouse/Partner | - | - |
| With Spouse/Partner & Roommate | -0.305 | - |
| Prefer Not to Answer/Don’t Know | -1.289 | - |
| *Note:* Hyphens refer to variable regression coefficients that were minimized to 0 by the LASSO optimization algorithm. | | |


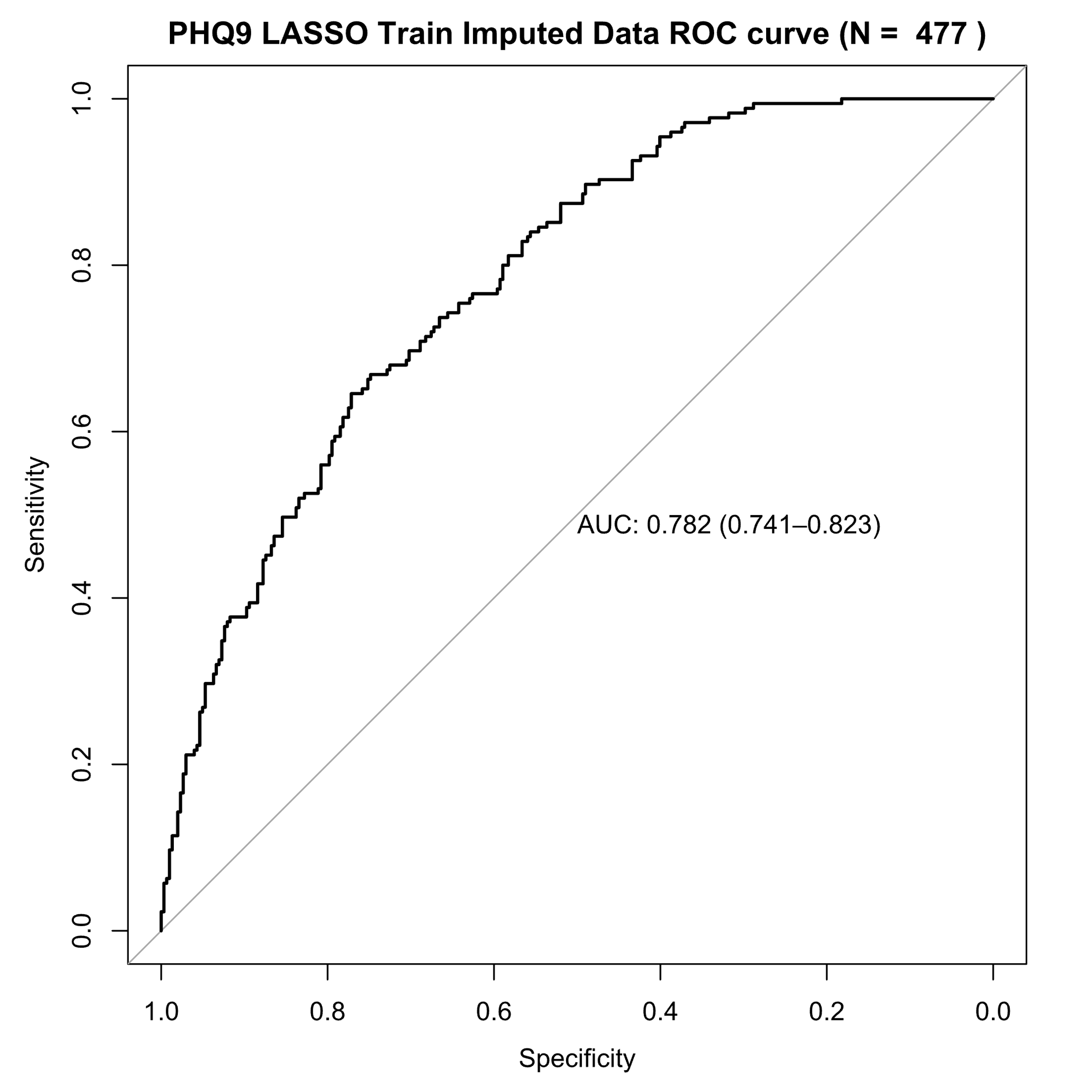


**Figure A1:** Receiver operating curve for predicting case complexity in training sample using PHQ-9 scores.


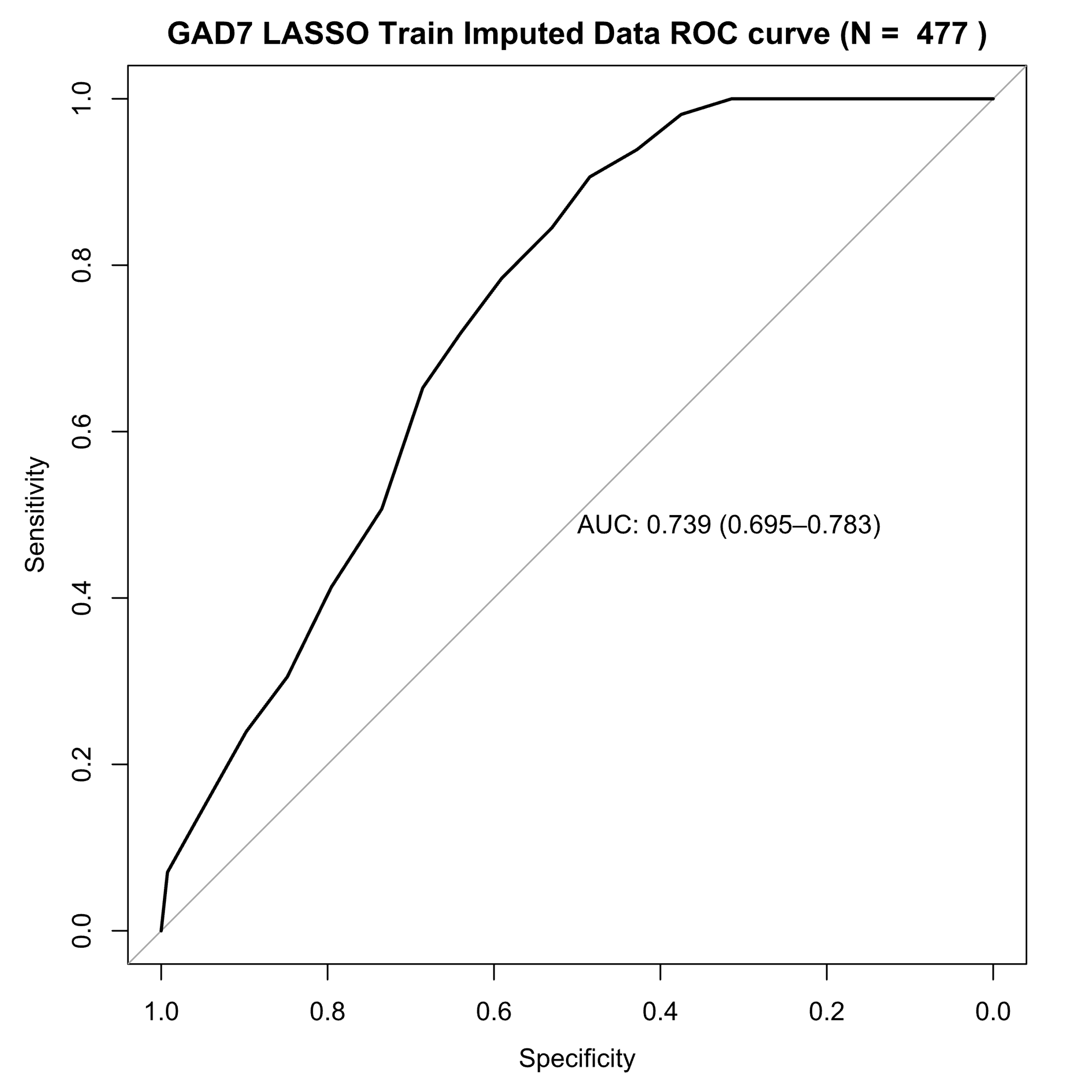


**Figure A2:** Receiver operating curve for predicting case complexity in training sample using GAD-7 scores.


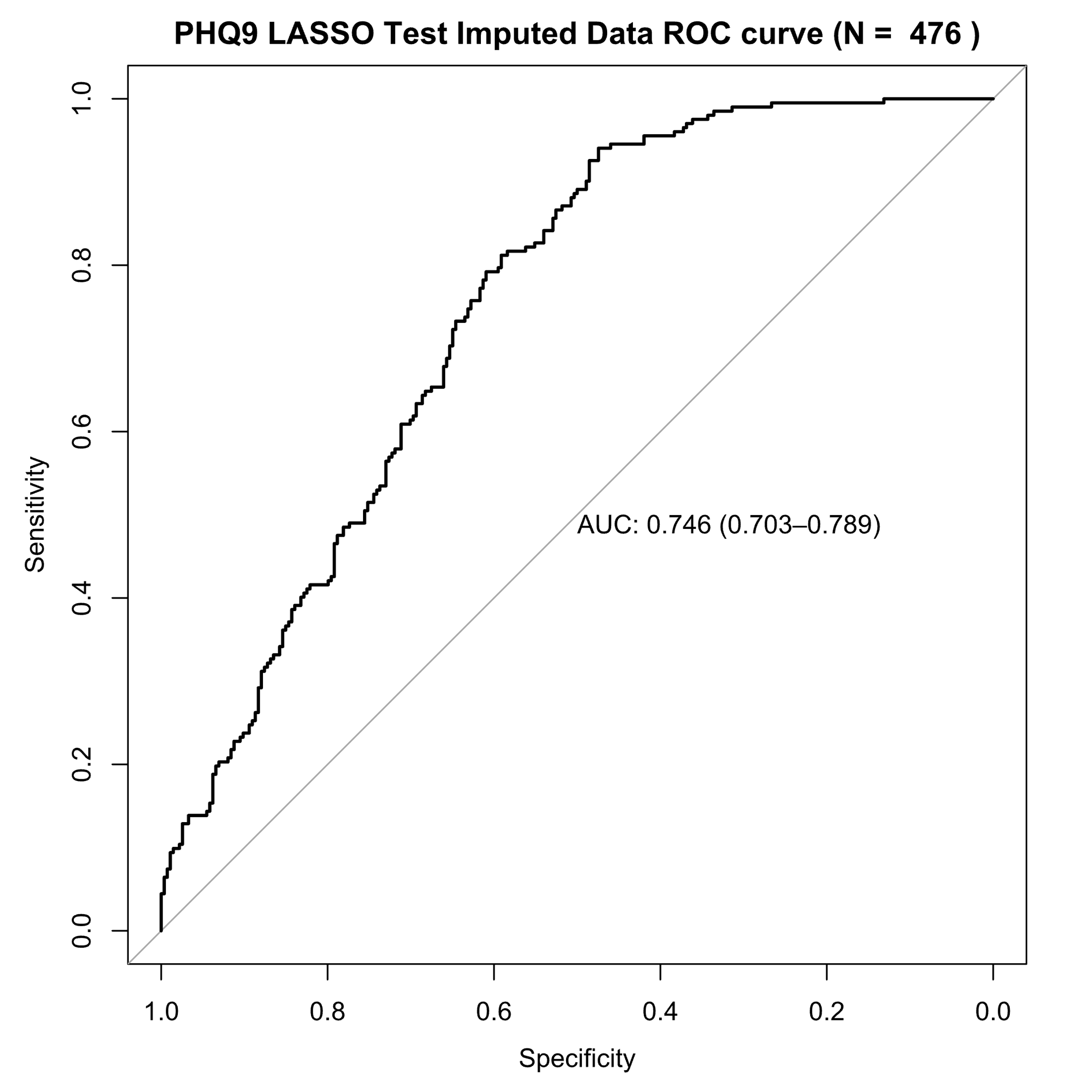


**Figure A3:** Receiver operating curve for predicting case complexity in validation test sample using PHQ-9 scores.


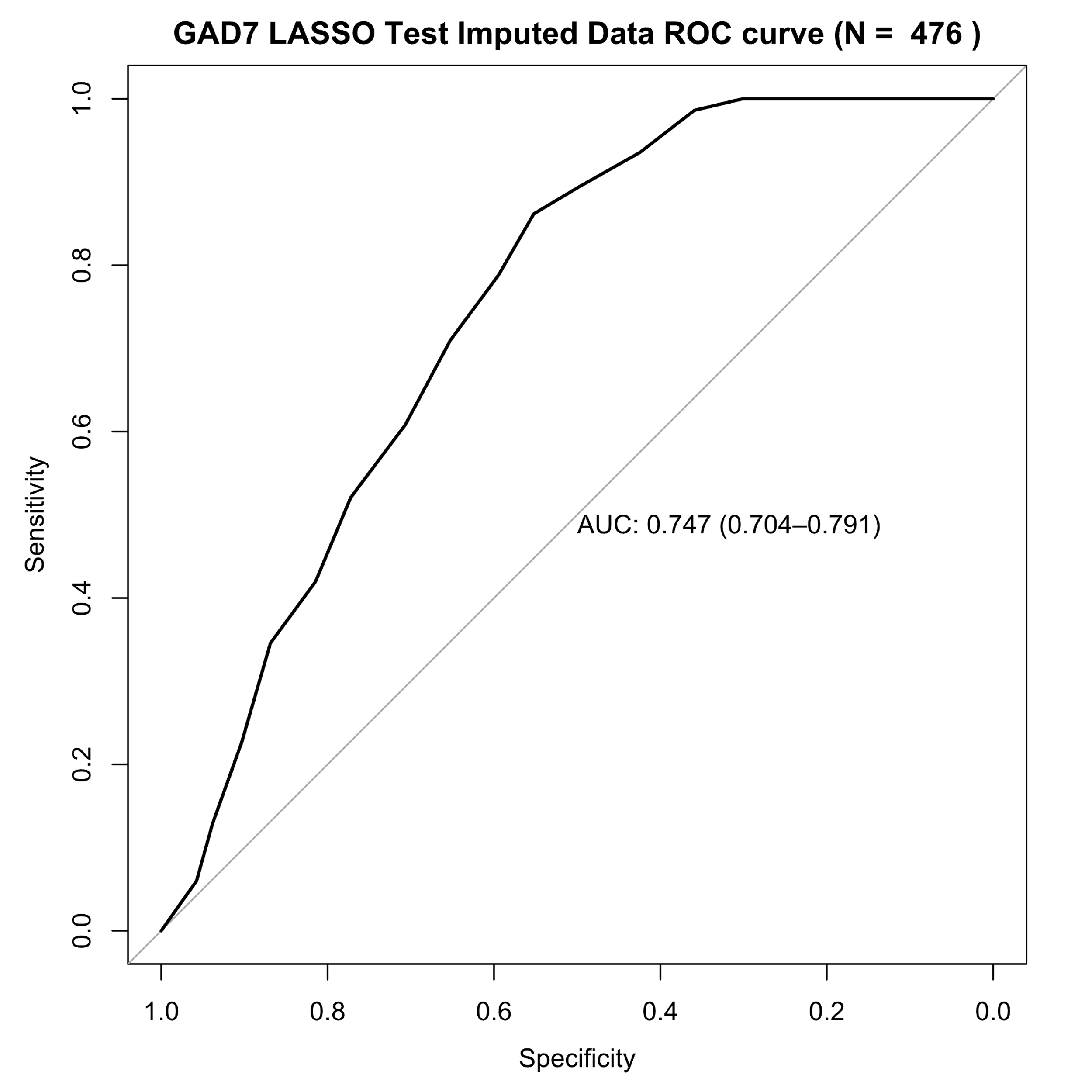


**Figure A4:** Receiver operating curve for predicting case complexity in validation test sample using GAD-7 scores.

**Table A3:** ORs comparing the intermediate PHQ-9 and GAD-7 LASSO predictive model predictions to Actual RCSI for PHQ-9 and GAD-7 respectively.

| Model | OR | OR 95% CI  (Upper, Lower) | Std. Error | Statistic | P-value |
| --- | --- | --- | --- | --- | --- |
| PHQ-0.5 | 3.15 | (2.14, 4.69) | 0.200 | 5.73 | < .00001 |
| PHQ-ROC | 6.06 | (3.99, 9.38) | 0.218 | 8.27 | < .00001 |
| GAD-0.5 | 3.68 | (2.49, 5.49) | 0.201 | 6.49 | < .00001 |
| GAD-ROC | 7.68 | (4.93, 12.30) | 0.233 | 8.75 | < .00001 |

**Table A4:** Confusion matrix performance metrics comparing ROC and 0.5 cut-off thresholds for the intermediate LASSO predictive models.

| *Model Type:* | **LASSO** | **LASSO** | **LASSO** | **LASSO** |
| --- | --- | --- | --- | --- |
| *RSCI Type:* | **PHQ-9** | **PHQ-9** | **GAD-7** | **GAD-7** |
| **Statistic** *Cut-off:* | **0.5** | **ROC** | **0.5** | **ROC** |
| Sensitivity | 0.490 | 0.812 | 0.521 | 0.862 |
| Specificity | 0.766 | 0.584 | 0.772 | 0.552 |
| Positive Predictive Value | 0.607 | 0.590 | 0.657 | 0.617 |
| Negative Predictive Value | 0.671 | 0.808 | 0.658 | 0.827 |
| False Positive Rate (Type I Error) | 0.234 | 0.416 | 0.228 | 0.448 |
| False Negative Rate (Type II Error) | 0.510 | 0.188 | 0.479 | 0.138 |
| Accuracy | 0.649 | 0.681 | 0.658 | 0.693 |
| Balanced Accuracy | 0.628 | 0.698 | 0.646 | 0.707 |
| F1 score | 0.542 | 0.683 | 0.581 | 0.719 |
